# Hypertrophic pachymeningitis IgG4-related with antineutrophil cytoplasmic antibody positivity and aortitis

**DOI:** 10.1093/rap/rkz006

**Published:** 2019-03-05

**Authors:** Alessia Musto, Anna Laura Fedele, Marco Gessi, Roberto Pallini, Fabrizio Cocciolillo, Angelo Zoli

**Affiliations:** 1 UOC of Rheumatology, Fondazione Policlinico Universitario A. Gemelli - Catholic University of the Sacred Heart, Rome, Italy; 2 UOC of Histopathology, Fondazione Policlinico Universitario A. Gemelli - Catholic University of the Sacred Heart, Rome, Italy; 3 UOC of Neurosurgery, Fondazione Policlinico Universitario A. Gemelli - Catholic University of the Sacred Heart, Rome, Italy; 4 UOC of Nuclear Medicine, Fondazione Policlinico Universitario A. Gemelli - Catholic University of the Sacred Heart, Rome, Italy

**Key message**

- ANCAs in IgG4-related disease might sustain an underlying vasculitic process.

Sir, IgG4-related disease (RD) is a rare and often misdiagnosed systemic disease characterized by diffuse/focal organ enlargement, mass-forming or nodular/thickened lesions in various organs and a prominent infiltration of lymphocytes and IgG4-positive plasmacytes with fibrosis [1]. Substantial overlap between IgG4-RD and ANCA-associated vasculitides (AAVs) exists in terms of organ involvement and histopathological features. Recent reports have raised the possibility that some patients with IgG4-RD are ANCA positive, thus suggesting that the role of ANCA in the diagnostic work-up should be reconsidered. There are also a few cases of concomitant biopsy-proven IgG4-RD and systemic involvement of AAV with a positive ANCA assay [2].

Hypertrophic pachymeningitis is a very rare manifestation of IgG4-RD. Only four cases of hypertrophic pachymeningitis IgG4-RD with a positive ANCA assay have been described in the literature, and one with pachymeningitis of the spine [2].

Here, we report a case of IgG4-RD involving the dura mater with a hypertrophic pachymeningitis and a positive ANCA assay with aortitis of the aortic arch. We also review the literature in order to provide clinicians with tools for interpreting ANCA positivity in IgG4-RD patients.

A 59-year-old woman was referred for an ingravescent headache and neck pain. A gadolinium-enhanced MRI of the brain disclosed a diffuse neoformed meningeal tissue at the level of the foramen magnum, with compression on the bulbo-medullary junction. Therefore, a suboccipital craniectomy and C1 laminectomy was performed, removing part of the tissue.

The patient’s serum tested positively for ANCA by both immunofluorescence (cytoplasmic ANCA pattern) and enzyme immunoassay, with specificity for PR3 (>860 a.u.; normal value <20 a.u.), with negative microbiological examinations for recent or past infections. Assays for ANA, RF, antibodies to ds DNA, thyroperoxidase and thyroglobulin were negative. The CRP was elevated (21 mg/l; normal <5 mg/l); ESR and tests of renal, thyroid and liver function were within the normal ranges.

The histopathological specimens consisted of dense fibrous dura mater tissue, with hypertrophic and chronic inflammatory features. The tissue was characterized by dense inflammatory infiltrates composed of CD3^+^ and CD8^+^ lymphocytes, CD138^+^ plasma cells and CD68^+^ macrophages/histiocytes. Focally, discrete follicular and microfollicular aggregates of CD20^+^ lymphocytes and scanty infiltrates of neutrophils were present. The immunohistochemical analysis revealed several IgG4^+^ plasma cells. No granulomatous or vasculitic features or fibrinoid necrosis of vessel walls could be detected.

**Fig. 1:**
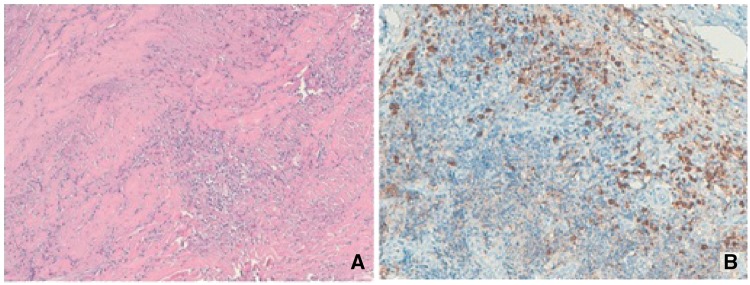
Neuropathological features of chronic hypertrophic meningitis (A) The neuropathological analysis showed dural tissue with hypertrophic features and chronic inflammation. (B, C) Many CD3^+^ lymphocytes and CD138^+^ plasma cells were present (B and C, respectively). (D) However, several IgG4^+^ plasma cells can be also found. [Haematoxylin and Eosin staining (A); CD3 (B), CD138 (C) and IgG4 (D) immunostaining, respectively.]

These histopatological findings were consistent with the suspicion of IgG4-related hypertrophic pachymeningitis (Fig. 1).

**Fig. 2:**
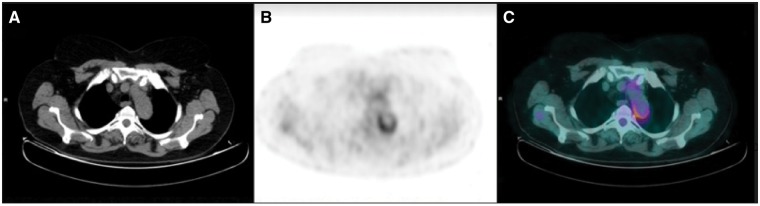
^18^F-Fluorodeoxyglucose PET/CT images FDG PET/CT images showed an area of increased radiopharmaceutical uptake in the aortic arch walls. (A) Axial CT slice. (B) Axial PET slice. (C) Axial fused images. FDG: fluorodeoxyglucose.

Thoracic and abdominal CT with iodinate contrast media excluded the presence of nodules and granulomatous lesions characteristic of parenchymal involvement in AAV, in addition to the presence of pancreatic involvement, retroperitoneal fibrosis or other organ-specific alterations typical of IgG4-RD. However, CT revealed a diffuse enhancement of the posterior wall of the aortic arch as a mural inflammation, with an intense ^18^F-fluorodeoxyglucose (^18^F-FDG) uptake on PET/CT study (Fig. 2). Treatment with prednisone (1 mg/kg) and rituximab induced swift remission of both IgG4-RD and AAV manifestations.

We identified 11 other reports of patients with IgG4-RD and positive ANCA in the literature; three with hypertrophic pachymeningitis IgG4-RD biopsy proved and positive ANCA, and one with IgG4-RD diagnosed presumptively.

The association between ANCA positivity with aortitis and IgG4-RD could suggest pathophysiological similarities between the two rare diseases. François-Xavier Danlos *et al.* [3] assume that the association between AAV and IgG4-RD is possible and represents a peculiar entity, with a particular phenotype and a good sensitivity to rituximab treatment. Several case reports have described chronic periaortitis in ANCA-positive patients [4], but histological samples were usually not available. Therefore, it was unclear whether such manifestations were attributable to vasculitis, granulomatous inflammation or predominant IgG4-RD-like pathology [5, 6]. A common pathophysiological pathway could involve T follicular helper (Tfh) cells, which were shown to have increased in both diseases and polarized towards the Tfh-2 subtype, enhancing IgG4-plasma cell polarization [7, 8]. However, the pathophysiology of AAV is complex and not clearly understood, as are the mechanisms of IgG4-RD, and further evidence is needed.

Our clinical case, in view of the coexistence of periaortitis and hypertrophic pachymeningitis with bioptic IgG4 infiltrates and serum ANCA positivity, seems to confirm this correlation between IgG4-RD and AAV, underlying the possibility of a new overlap syndrome, as postulated in the study by Danlos *et al.* [3]; however, it deserves a larger case series and a longer follow-up.

*Funding*: No specific funding was received from any bodies in the public, commercial or not-for-profit sectors to carry out the work described in this manuscript.

*Disclosure statement*: The authors have declared no conflicts of interest.
